# Artificial Intelligence as an Aid in CBCT Airway Analysis: A Systematic Review

**DOI:** 10.3390/life12111894

**Published:** 2022-11-15

**Authors:** Ioannis A. Tsolakis, Olga-Elpis Kolokitha, Erofili Papadopoulou, Apostolos I. Tsolakis, Evangelos G. Kilipiris, J. Martin Palomo

**Affiliations:** 1 Department of Orthodontics, School of Dentistry, Aristotle University of Thessaloniki, 54124 Thessaloniki, Greece; 2 Department of Oral Medicine & Pathology and Hospital Dentistry, School of Dentistry, National and Kapodistrian University of Athens, 10679 Athens, Greece; 3 Department of Orthodontics, School of Dentistry, National and Kapodistrian University of Athens, 10679 Athens, Greece; 4 Department of Orthodontics, Case Western Reserve University School of Dental Medicine, Cleveland, OH 44106, USA; 5 Department of Maxillofacial Surgery, F.D. Roosevelt University Hospital, 97517 Banska Bystrica, Slovakia

**Keywords:** Artificial Intelligence, CBCT, airway

## Abstract

Background: The use of artificial intelligence (AI) in health sciences is becoming increasingly popular among doctors nowadays. This study evaluated the literature regarding the use of AI for CBCT airway analysis. To our knowledge, this is the first systematic review that examines the performance of artificial intelligence in CBCT airway analysis. Methods: Electronic databases and the reference lists of the relevant research papers were searched for published and unpublished literature. Study selection, data extraction, and risk of bias evaluation were all carried out independently and twice. Finally, five articles were chosen. Results: The results suggested a high correlation between the automatic and manual airway measurements indicating that the airway measurements may be automatically and accurately calculated from CBCT images. Conclusions: According to the present literature, automatic airway segmentation can be used for clinical purposes. The main key findings of this systematic review are that the automatic airway segmentation is accurate in the measurement of the airway and, at the same time, appears to be fast and easy to use. However, the present literature is really limited, and more studies in the future providing high-quality evidence are needed.

## 1. Introduction

The digital era in health sciences has been ushered in by recent innovations like cone beam computed tomography (CBCT), 3D printing, and artificial intelligence (AI). Those innovations have been playing an important role in the field of health sciences to support diagnosis and customized treatment solutions [1,2,3]. In 1956, Dartmouth University was the first to use the terminology “artificial intelligence”, which is defined as computerized synthetic human cognitive function [4]. Since then, the application of AI has grown dramatically [5,6]. Thanks to improvements in analytics methods, computing power, and data accessibility, AI may touch many aspects of modern culture. On a global scale, we are already noticing its effects on our day-to-day lives. In addition to filtering information for social media and web searches, it also does this for consumer electronics like cameras, cellphones, tablets, and even cars. Due to the scientific method, which is presently undergoing a paradigm shift in the multidisciplinary link between AI and data science, all recent advancements and advances in dentistry have been made possible [7,8]. 

Artificial intelligence (AI) refers to fundamental technologies including deep learning, artificial neural networks (ANNs), and machine learning. A significant area of artificial intelligence is machine learning. Machine learning makes predictions about new data and circumstances using the statistical patterns of previously learned data. Training data are necessary for machine learning to function. Machine learning cannot work without training data. With this approach, the computer model can develop over time by learning from experience rather than through conventional explicit programming. A model needs a large quantity of data to using abstractions from various processing levels. A subset of artificial intelligence is deep learning. Deep learning is the process by which computers learn to think utilizing structures inspired by the human brain, whereas machine learning is the process by which computers learn to think and act with less human intervention. The benefit of deep learning is that little engineering effort is needed to prepare the data for analysis. Recognition of visual objects and item identification have seen the most use of deep learning techniques. Orthodontic and Otolaryngology (ORL) clinical applications favor more advanced AI solutions, such as cone beam computed tomography (CBCT) and 3D convolutional neural networks (3D CNN). “Strong AI” or “deep AI” is a type of artificial general intelligence (AGI) that is comparable to humans in terms of problem-solving ability. In contrast to the majority of today’s advanced AI algorithms, physicians possess the capacities for abstract thought, strategic planning, and the generation of original ideas [9]. AGI will have the capacity to think very much like a human. Beyond our comprehension, artificial super intelligence (ASI) will be able to learn and grow beyond human capacities.

A notable dilemma involving legal culpability for flaws in AI algorithm assessment and potential erroneous medical intervention is one of the issues surrounding AI application. As is well known, AI can be programmed to reflect the biases of any individual [10]. We do not know how deep AI algorithms generated the results, which is another dilemma that Zhang et al. addressed [11]. Due to the black-box nature of AI processes, current research has focused on “explainable artificial intelligence (XAI)” to get around this limitation. In contrast to AI methods like deep learning, XAI may offer both decision-making and model explanations [11]. Several publications cover this subject [12,13].

The upper airway, also known as the pharyngeal airway space, is a complicated anatomical region that is closely related to the nearby bone and soft tissue components. It is mostly in charge of carrying out actions including breathing, speaking, and swallowing [14]. Since multiple investigations have shown its connection to craniofacial growth and development, the upper airway assessment has attracted the attention of physicians [15,16,17,18]. Since the craniofacial complex could be responsible for possible constrictions of the upper airway, physicians used surgical and non-invasive methods to change the anatomy and resolve the airway constriction. The use of X-rays is really important to assess the effectiveness and find the possible side effects of these treatments [19]. In the past, two-dimensional (2D) lateral cephalometry was used to evaluate airway alterations in patients with dentofacial and skeletal anomalies during the stages of diagnosis, treatment planning, and follow-up [20,21]. However, due to the possible drawbacks of 2D approaches to representing the upper airway, computed tomography (CT), cone-beam CT (CBCT), and magnetic resonance imaging (MRI) have largely taken their position as a clinical standard for assessing upper airway volume and dimensional changes in order to comprehend their pathogenesis [22,23,24]. CBCT has proven to be as accurate as other gold standard methods for measuring the upper airway volume and constricted area [25].

It is now proved that CBCT can be used in every day practice to accurately measure the airway volume and minimum cross-section area; this plays a critical role in evaluating and managing different airway disorders [25]. The use of artificial intelligence in CBCT airway analysis could provide accurate and fast measurements to the clinicians. This would be translated to faster management of different airway disorders that could be life threatening. This article aims to systematically review the current literature on the use of artificial intelligence in CBCT airway analysis. To our knowledge, this is the first systematic review that examines the performance of artificial intelligence in CBCT airway analysis. 

## 2. Materials and Methods

### 2.1. Protocol and Registration

The protocol for this present systematic review was registered on the Open Science Forum Database following the Prisma-P guidelines1 (Protocol: 10.17605/OSF.IO/4HWBJ).

This systematic review was conducted by using the following keywords in the search strategy “Artificial Intelligence”, “airway volume”, “cbct”. Those keywords were com-bined with the following Medical Subject Heading (MeSH terms): “Artificial Intelligence” [Mesh], “Cone-Beam Computed Tomography” [Mesh]. The databases used for the electronic search were Med-line (PubMed), Cochrane Library, and Scopus. A manual search was also carried out. There was a choice of exclusively English-written papers, and the publication duration was unrestricted. Personal opinions were omitted from studies. The search was conducted for studies published until July 2022. The search strategy for PubMed is presented in Table 1.

Studies were chosen by three authors separately and in duplication (I.A.T., E.P., E.G.K.). Discussion with other authors helped to clarify any potential inconsistencies (O.K., A.I.T, J.M.P). The names of the studies’ authors, their institutions, or their research conclusions were revealed (not blinded). The authors first looked for possibly pertinent research by title, then they read the abstract and eliminated any irrelevant papers. Later, to locate more papers that were missed by database searches, a manual search of relevant study references was conducted. Finally, after thoroughly reviewing all of the papers, a decision was taken based on our inclusion and exclusion criteria (Table 2).

### 2.2. Data Items and Collection Extraction and Management

The data were independently extracted and duplicated by three review writers (I.A.T., E.P., E.G.K.). Study participants, the intervention, the results, the techniques of outcome evaluation, the findings, and the conclusion were among the data that were extracted. The present authors reported and examined only the data that were available because they did not have access to any missing data.

### 2.3. Risk of Bias/Quality Assessment in Individual Studies

The Cochrane Quality Assessment of the ACROBAT-NRSI tool was used to evaluate the methodology of the included studies and determine whether there were any applicability or bias problems. Based on the following, each domain was evaluated and classified as high risk, low risk, or unclear:

1. Low risk of bias if all key domains of the study were at low risk of bias.
2. Unclear risk of bias if one or more key domains of the study were unclear.
3. High risk of bias if one or more key domains were at high risk of bias.

## 3. Results

In the initial data search, 1050 studies were found from all data bases. Only 70 of these papers were chosen, based on the study’s title. Each chosen article was then thoroughly assessed by three independent authors who read the complete document. Five publications in all were chosen for the present systematic review. Four studies were the result of PubMed search while on extra research paper was found through Scopus. 

**Figure 1:**
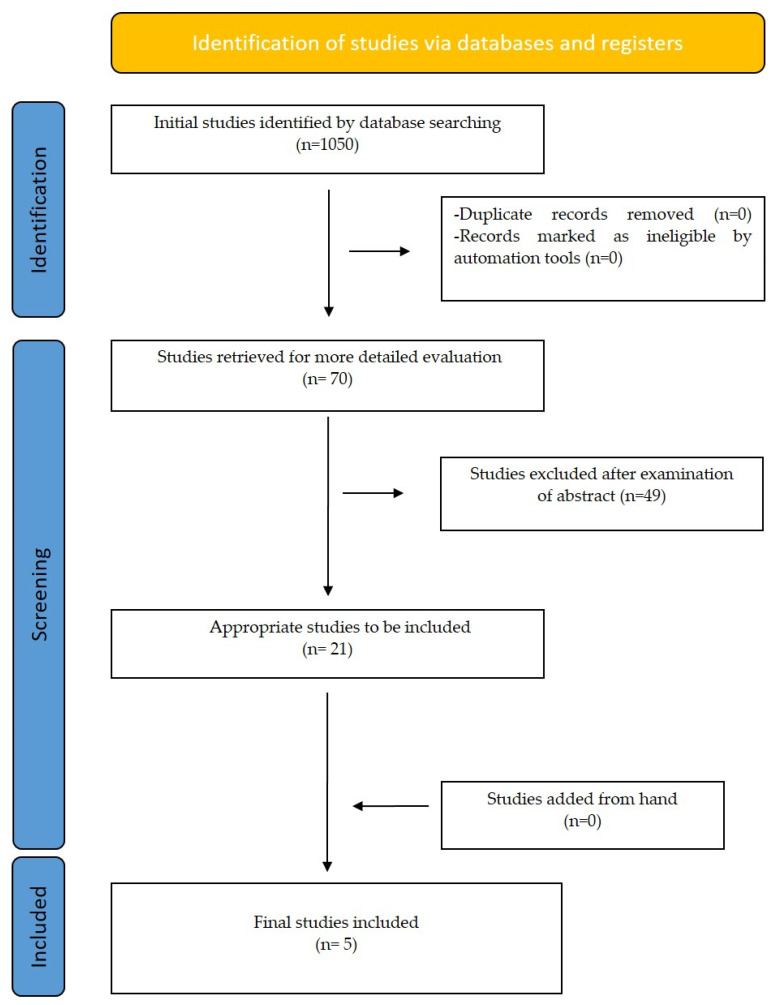
Prisma Flow diagram–selection of studies.

All the final selected articles were prospective studies. All studies evaluated the accuracy of AI systems in segmenting and calculating airway volume based on CNN and RNN models. Three articles used their own model for software usage while one of the remaining two used the Mimics 19.0, InVivo 5 software and the other one the Diognocat, InVivo 5 software [26,27,28,29,30]. The procedure of article selection is presented on a flow diagram (Figure 1), and data are briefly presented in Table 3.

### Risk of Bias within Studies

The following seven criteria were applied to non-RCT studies: bias due to confounding, bias in the selection of participants into the study, bias in the measurement of interventions, bias due to departures from intended interventions, bias due to missing data, bias in measurement outcomes and bias in the selection of the reported result. Two of the studies presented high risk of bias while the other three presented low risk of bias in all measurements (Table 4).

## 4. Discussion

All studies in the present systematic review reported equal conclusions regarding the accuracy and the benefit of automatic segmentation of the upper airway by deep learning methods. It is important to mention that all studies were reported in 2021 and after. This shows that the scientific interest in AI and airway volume segmentation has really increased in the last few years. We were able to access all the known databases to minimize the limitations of this study. However, a possible limitation could appear for articles not included in any of the databases we used. 

The first and most important stage in assessing the upper airway volume is segmentation, which allows the airway space to be distinguished from the rest of the scan and can then be visualized and quantified in three dimensions. Various upper airway segmentation methods and algorithms that are either manual, semiautomatic, or automatic in nature have been developed over the past ten years [31]. Even while hand segmentation is the gold standard and provides the most precise replication of the anatomical structure, it is labor- and time-intensive. Numerous threshold-based semiautomatic software programs have been approved for volumetric assessment as well [32]. In these programs, the user specifies a volume of interest (VOI), and the program automatically combines the gray threshold values in that area without effectively taking into account the image intensity and anatomical variations. Similar to this, several studies have proposed a set threshold value for segmenting the upper airway [33,34], although this value may change based on the CBCT equipment, scanning parameters, machine calibration, and noise from patient movement or metal artifacts [35,36]. The semiautomatic method has proven to be accurately measured by different software [37]. For the segmentation of the upper airway, prior studies have also presented fully automatic advanced and hybrid picture segmentation techniques. However, due to either poor precision, fixed thresholding, manual localization of seed points, manual VOI selection, reliance on picture orientation, or algorithmic failure under varied scanning parameters, they are of limited value [32,38,39,40].

The first study discussed here was reported in early 2021 from Leonardi et al. Aiming to fully automate the segmentation of the pharyngeal airway, and the sinonasal cavity from cone-beam computed tomography (CBCT) scans, they evaluated the accuracy of a new autonomous deep learning-based approach. In order to manually segment the sinonasal cavity and pharyngeal subregions of 40 healthy patients’ CBCT scans (20 women and 20 men), Mimics software was used (version 20.0; Materialise, Leuven, Belgium). A total of 20 CBCT scans were chosen at random from the entire sample and used to train the AI model file. By contrasting the segmentation volumes of the 3D models created with automatic and manual segmentations, the remaining 20 CBCT segmentation masks were utilized to assess the precision of the CNN completely automatic technique. Their results suggested a low model error (0.31 mm^3^), and all the measurements were highly correlated with an intraclass correlation coefficient of 0.921. Between the approaches, there was a mean difference of 1.93 ± 0.73 cm^3^, but it was not statistically significant (*p* > 0.05). The average matching percentage found was 85.35 ± 2.59 and 93.44 ± 2.54. The disparities between the assessments made using the two approaches were 3.3% and 5.8%, respectively, as expressed by the Dice score coefficient in percentage [26].

In April of 2021, Park et al. reported on the precision of an airway volume measurement made using a deep learning model based on regression neural networks. The system for entirely automatic segmentation of a deep learning process was built using a set of manually drawn airway data. One examiner used the mid-sagittal plane of 315 patients’ cone-beam computed tomography (CBCT) scans to identify the manual landmarks of the airway. They performed clinical dataset-based training with data augmentation. The airway channel was measured and segmented using markers that were annotated. The authors were able to verify the accuracy of the model by assessing the following differences between the examiner and the program: (1) a difference in the nasopharynx, oropharynx, and hypopharynx volume; and (2) the Euclidean distance. A total of 61 samples were collected and compared for the agreement analysis. The correlation test revealed a reliability range from high to outstanding. Regression analysis was used to examine differences in volume. There was a strong linear regression connection between the slopes of the two measurements, which was close to 1 (r^2^ = 0.975, slope = 1.02, *p* < 0.001). These findings suggested that fully automatic airway segmentation is trainable using deep learning in artificial intelligence. Furthermore, they found that there was a strong correlation between manual data and deep learning data [27].

A month later, and more specifically in May of 2021, Shujaat et al. reported an evaluation of a deep learning-based three-dimensional (3D) convolutional neural network (CNN) model for automatically segmenting the pharyngeal airway space, and its performance was examined. From a database of individuals undergoing orthognathic surgery, 103 CT and CBCT scans were obtained. Two CBCT devices (Promax 3D Max, Planmeca, Helsinki, Finland, and Newtom VGi evo, Cefla, Imola, Italy) and one CT (128-slice multi-slice spiral CT, Siemens Somatom Definition Flash, Siemens AG, Erlangen, Germany) with various scanning parameters made up the acquisition devices. The airway was automatically segmented using a 3D CNN-based model called the 3D U-Net. The entire CT/CBCT dataset was divided into three sets: training set (*n* = 48) for training the model based on the observer-based manual segmentation that provided the basis for it; test set (*n* = 25) for obtaining the model’s final performance; and validation set (*n* = 30) for comparing the model’s performance to that of observer-based segmentation. Their results suggested that the segmented region could be distinguished by the CNN model with the best precision (0.97 ± 0.01) and recall (0.96 ± 0.03). The maximum deviation between the ground truth and the artificial segmentation based on the 95% Hausdorff distance score was 0.98 ± 0.74 mm. The segmented region’s high likeness to the real world was validated by the dice score of 0.97 ± 0.02. It was also discovered that the Intersection over Union (IoU) metric had a high value (0.93 ± 0.03). In comparison to the Promax 3D Max and CT device, the Newtom VGi Evo CBCT performed better based on the acquisition devices [28].

In December of 2021, Sin et al. reported on a deep learning artificial intelligence (AI) system to assess an automatic segmentation algorithm for the pharyngeal airway in cone-beam computed tomography (CBCT) images. In this retrospective investigation, data from 306 participants on the pharyngeal airway were included after archives of CBCT pictures were reviewed. Using serial CBCT images, a machine learning method built on Convolutional Neural Networks (CNN) segmented the pharyngeal airway. The airway was manually generated using semi-automatic software (ITK-SNAP), and the outcomes were contrasted with those of artificial intelligence. When comparing the results of measurements made by humans versus algorithms powered by artificial intelligence, the dice similarity coefficient (DSC) and intersection over union (IoU) were utilized as measures of segmentation accuracy. The average pharyngeal airway volume, according to the human observer, was 18.08 cm^3^, while the average volume of artificial intelligence was 17.32 cm^3^. It is possible to segment the pharyngeal airways with a dice ratio of 0.919 and a weighted IoU of 0.993 [29].

Finally, the study of Orhan et al. (2022) was characterized by two goals. The first goal of this work was to develop and verify an algorithm for automatically detecting the pharyngeal airway on CBCT data using artificial intelligence (AI) software called Diacat. The second goal was to compare the recently created artificial intelligence system to commercially available software for 3D CBCT evaluation in order to validate it. The pharyngeal airway in obstructive sleep apnea (OSA) patients was automatically assessed for the first time in this study. The segmentation of the pharyngeal airways in OSA and non-OSA patients was performed using a machine learning technique based on convolutional neural networks. Radiologists manually determined the airway using semi-automatic software, and their measurements were compared with those of the AI. The mean airway volumes of the several OSA patient groups (minimal, mild, moderate, and severe) were compared. In addition, patients with OSA and those without it were compared in terms of their airway’s narrowest points (mm), its field (mm^2^), and its volume (cc). In all groups, there was no statistically significant difference between measures taken using the manual method and those taken using the Diagnocat (*p* > 0.05). For manual and automated segmentation, the interclass correlation coefficients were 0.954 for Diagnocat, and 0.956 for automatic segmentation. They examined the output images to determine why the mean value for the total airway was higher in the DC measurement, even though there was no statistically significant difference in total airway volume measurements between the manual measurements, automatic measurements, and DC measurements in non-OSA and OSA patients. Due to the low soft-tissue contrast in CBCT images, it was shown that the DC algorithm also assesses the epiglottis volume and the posterior nasal aperture volume, which results in greater values for airway volume measurement [30].

According to this systematic review the present literature is really limited as regards studies that looked at the accuracy of artificial intelligence use for CBCT upper airway analysis. In order to have strong evidence on how accurately CBCT airway analysis is performed with the use of artificial intelligence, more randomized prospective studies should be performed. Those studies should limit all kind of bias in order to provide high quality evidence. 

## 5. Conclusions

The pharyngeal airway may now be automatically segmented from CBCT images thanks to a successful AI algorithm. According to the present literature, the automatic segmentation can be put to clinical use. This is because it appears to be accurate in the measurement of the airway but at the same time it appears to be fast and easy to use. However, there were only 5 studies in the present literature to support these data and only 3 of them reported a low risk of bias. More studies in the future providing high-quality evidence are needed.

## Figures and Tables

**Table 1:** The search strategy for PubMed.

“Cone-Beam Computed Tomography” [Mesh] AND airway volume	330 results
“Cone-Beam Computed Tomography” [Mesh] AND Artificial Intelligence” [Mesh]	257 results
Artificial Intelligence” [Mesh] AND airway volume	76 results
“Cone-Beam Computed Tomography” [Mesh] AND Artificial Intelligence” [Mesh] AND airway volume	4 results

**Table 2:** Inclusion and Exclusion criteria.

Inclusion Criteria	Exclusion Criteria
Studies that refer to the use of artificial intelligence for CBCT airway analysis	Studies that are reviews or authors’ opinion
prospective or retrospective studies	

**Table 3:** Data extraction.

Authors/ Publication Year	Study Design	Participants (Number of CBCT)	Intervention	Outcomes	Method of Outcome Assessment	Results	Authors/ Publication Year
Leonardi R et al. [26] (2021)	prospective	40 CBCT scans	1 CBCT device was used.	Accuracy of the CNN fully automatic segmentation of the sinonasal cavity pharyngeal airway	- Mimics software (version 22.0, materialize N.V., Leuven, Belgium). - Surface-to-surface matching technique.	Measurements were highly correlated with an intraclass correlation coefficient value of 0.921, whereas the method error was 0.31 mm^3^. A mean difference of 1.93 ± 6 ± 0.73 mm^3^ was found to be not statistically significant. The differences, measured as the Dice score coefficient in percentage, between the assessments done with both methods were 3.3% and 5.8%, respectively.	The new deep learning–based method for automated segmentation is accurate for airway segmentation
Park et al. [27]	prospective	315 CBCT scans	1 CBCT device was used.	Accuracy of the airway volume measurement by a Regression Neural Network-based deep-learning model	- MATLAB 2020a (MathWorks, Natick, MA, USA)	The total volume was the most correlated intra-class correlation coefficient (ICC) value in the oropharynx (0.986), followed by the hypopharynx (0.964), and the nasopharynx (0.912). The slope of the two measurements was close to 1 and showed a linear regression correlation (r^2^ = 0.975, slope = 1.02, p < 0.001).	These results indicate that fully automatic segmentation of the airway is possible by training via deep learning of artificial intelligence.
Shujaat S et al. [28] (2021)	prospective	103 CT and CBCT scans	Scans from 1 CT and 2 CBCTs were grouped in: - training set - test set - validation set	The performance of deep learning based 3D CNN model for automatic segmentation of the pharyngeal airway space	- Mimics software (version 22.0, materialize N.V., Leuven, Belgium).	The CNN model was able to identify the segmented region with optimal precision and recall. The maximal difference between the automatic segmentation and ground truth was 0.98 ± 0.74 mm. The dice score of 0.97 ± 0.02 confirmed the high similarity of the segmented region to the ground truth.	The proposed 3D U-Net model offered an accurate method for the segmentation of Airway from CT/CBCT images.
Sin Ç et al. [29] (2021)	prospective	306 CBCT scans	1 CBCT device was used and grouped in: - training, - validation - test sets	The accuracy of an automatic detection algorithm for pharyngeal airway on CBCT images using a deep-learning artificial intelligence system	- Open-source version 3.8 ITK SNAP software - MATLAB implementation of U-Net and SGD Adam optimizer.	There was no statistically significant difference between the human observation of the average volume of the pharyngeal airway and the results from artificial intelligence The ICC between researcher and AI measurements was found to be highly correlated (0.985) The calculated Dice ratio across all slices of all CBCT images was 0.919, and the mean accuracy of 0.961 providing excellent accuracy.	AI models based on deep learning techniques can be used for easy and error-free segmentation of pharyngeal airway volume from CBCT
Orhan K et al. [30] (2022)	prospective	200 CBCT scans	3 CBCT devices	To validate an automatic detection algorithm for pharyngeal airway on CBCT data using an AI software for OSA patients To validate the newly developed artificial intelligence system in comparison to commercially available software for 3D CBCT evaluation.	- Diagnocat, InVivo 5	• There was no statistically significant difference between the manual technique and Diagnocat measurements in all groups (p > 0.05). • Inter-class correlation coefficients were 0.954 for manual and automatic segmentation, 0.956 for Diagnocat and automatic segmentation, 0.972 for Diagnocat and manual segmentation. • It was seen that the DC algorithm also measures the epiglottis volume and the posterior nasal aperture volume due to the low soft-tissue contrast in CBCT images; this leads to higher values in airway volume measurement.	Activating this potential collaboration for OSA patients would significantly reduce the effort and time required for the initial diagnosis and follow-up of these patients.

**Table 4:** Risk of bias assessment.

Author (Year)	Outcomes	Bias Due to Confounding	Bias in Selection of Participants into the Study	Bias in Measurement of Interventions	Bias Due to Departures from Intended Interventions	Bias Due to Missing Data	Bias in Measurement of Outcomes	Bias in Selection of the Reported Result	Overall Bias
Leonardi R et al. [26] (2021)	Accuracy of the CNN fully automatic segmentation of the airway	Low for all outcomes	Low for all outcomes	Low for all outcomes	Low for all outcomes	Low for all outcomes	High for all outcomes	Low for all outcomes	High for all outcomes
Park et al. [27] (2021)	Accuracy of the airway volume measurement by a Regression Neural Network	Low for all outcomes	Low for all outcomes	Low for all outcomes	Low for all outcomes	Low for all outcomes	High for all outcomes	Low for all outcomes	High for all outcomes
Shujaat S et al. [28] (2021)	Accuracy of 3D CNN model for automatic segmentation of the pharyngeal airway space	Low for all outcomes	Low for all outcomes	Low for all outcomes	Low for all outcomes	Low for all outcomes	Low for all outcomes	Low for all outcomes	Low for all outcomes
Sin Ç et al. [29] (2021)	Accuracy of an automatic detection algorithm for pharyngeal airway	Low for all outcomes	Low for all outcomes	Low for all outcomes	Low for all outcomes	Low for all outcomes	Low for all outcomes	Low for all outcomes	Low for all outcomes
Orhan K et al. [30] (2022)	To validate an automatic detection algorithm for pharyngeal airway with AI for OSA patients	Low for all outcomes	Low for all outcomes	Low for all outcomes	Low for all outcomes	Low for all outcomes	Low for all outcomes	Low for all outcomes	Low for all outcomes

## Data Availability

Not applicable.
